# PEDV and PDCoV Pathogenesis: The Interplay Between Host Innate Immune Responses and Porcine Enteric Coronaviruses

**DOI:** 10.3389/fvets.2019.00034

**Published:** 2019-02-22

**Authors:** Surapong Koonpaew, Samaporn Teeravechyan, Phanramphoei Namprachan Frantz, Thanathom Chailangkarn, Anan Jongkaewwattana

**Affiliations:** Virology and Cell Technology Laboratory, National Center for Genetic Engineering and Biotechnology (BIOTEC), National Science and Technology Development Agency, Pathumthani, Thailand

**Keywords:** PEDV, PDCoV, innate antiviral response, interferon induction and signaling, innate immune antagonism

## Abstract

Enteropathogenic porcine epidemic diarrhea virus (PEDV) and porcine deltacoronavirus (PDCoV), members of the coronavirus family, account for the majority of lethal watery diarrhea in neonatal pigs in the past decade. These two viruses pose significant economic and public health burdens, even as both continue to emerge and reemerge worldwide. The ability to evade, circumvent or subvert the host’s first line of defense, namely the innate immune system, is the key determinant for pathogen virulence, survival, and the establishment of successful infection. Unfortunately, we have only started to unravel the underlying viral mechanisms used to manipulate host innate immune responses. In this review, we gather current knowledge concerning the interplay between these viruses and components of host innate immunity, focusing on type I interferon induction and signaling in particular, and the mechanisms by which virus-encoded gene products antagonize and subvert host innate immune responses. Finally, we provide some perspectives on the advantages gained from a better understanding of host-pathogen interactions. This includes their implications for the future development of PEDV and PDCoV vaccines and how we can further our knowledge of the molecular mechanisms underlying virus pathogenesis, virulence, and host coevolution.

## Introduction

Two members of swine enteric coronaviruses, porcine epidemic diarrhea virus (PEDV), and porcine deltacoronavirus (PDCoV), have recently emerged as major causative agents of lethal watery diarrhea in piglets, leading to significant losses within the swine industry worldwide. PEDV and PDCoV are classified in distinct genera in the family *Coronaviridae*, as an *Alphacoronavirus* and *Deltacoronavirus*, respectively (1, 2). The transmissible gastroenteritis virus (TGEV), also an enteropathogenic porcine alphacoronavirus, used to be responsible for severe economic losses around the globe in the 1990s. However, due to its current disappearance in many parts of the world, this review will focus mainly on PEDV and PDCoV, the two emerging swine coronaviruses.

The first PEDV outbreak occurred in Europe around 1970s (3, 4). From the 1990s onward, sporadic occurrences of PEDV infection were reported in countries such as the Czech Republic, Belgium, Hungary, South Korea, China, Italy, and Thailand (5) before emerging as a major swine outbreak in China around 2010 (6, 7). This outbreak marked the appearance of highly pathogenic strains of PEDV associated with 80–100% morbidity and 50–90% mortality in suckling piglets (8). 2013 was another critical year, seeing the emergence of PEDV in the North American continent (9). More recently, the epidemiology of PEDV has taken a new turn, with China seeing increasing co-infection rates (up to 51%) with PDCoV (10, 11).

Compared to the discovery of PEDV, the first report of PDCoV was fairly recent, being detected in 2012 in Hong Kong during molecular surveillance of coronaviruses in avian and mammalian species (2). To date, PDCoV has been detected in many countries including the United States, Canada, South Korea, China, Thailand, Laos, and Vietnam (12, 13). Clinical severity of PDCoV infection tends to be lower than PEDV, with a mortality rate of around 40% when experimentally inoculated into gnotobiotic suckling piglets (14, 15). Nevertheless, PDCoV still causes severe disease (16). Among diarrheic pigs in the United States and China, the prevalence of PDCoV was found to be as high as 30–7%, respectively of all reported cases (10). Accordingly, PDCoV is an emerging pathogen that warrants further study because there is still little information about deltacoronavirus infection, pathogenesis, and virus-host interaction (17).

Innate immunity functions as the first line of defense against invading viruses. It identifies and alerts host cells to their presence by eliciting rapid and early cellular responses and inducing production of multiple cytokines. Lymphoid-associated tissues (including Peyer’s patches, lymphoid follicles, and mesenteric lymph nodes) are the largest and the first barrier against infections of the gastrointestinal (GI) tract (18). Gut-associated lymphoid tissue (GALT)-resident professional antigen presenting cells (APCs) are therefore of particular interest in studying PEDV and PDCoV infection, with APCs such as dendritic cells (DCs) most prominently plasmacytoid DCs (pDCs) which are the major producers of types I interferons (IFNs) *in vivo* during viral infection (19) and macrophages being the first immune cells to encounter PEDV, PDCoV, and other enteric viruses (20).

Enteric coronaviruses possess pathogen-associated molecular patterns (PAMPs) such as viral glycoprotein structures and viral RNAs which can be recognized by pattern recognition receptors (PRRs) present on APCs (21). Recognition events initiate propagation of intracellular signaling, resulting in production of soluble antiviral components of innate immunity. These soluble components are primarily made up of type I and III IFNs, chemokines, and proinflammatory cytokines. Because the IFN pathway is crucial in initiating viral resistance and shaping subsequent adaptive immune responses (22), both PEDV and PDCoV need to evolve mechanisms to antagonize and suppress its induction and signaling in order to establish productive infection. Innate immune cell populations such as natural killer (NK) cells are also known to respond to porcine coronavirus infections and may play a role in disease outcome and pathogenesis (23).

In the following sections, we will describe the relevant aspects of PEDV and PDCoV biology and pathogenesis, and review the fundamentals of antiviral innate immunity. Subsequent sections will provide an update on recent studies regarding host antiviral innate responses as well as key mechanisms and strategies that these porcine enteric coronaviruses have evolved to evade virus recognition by host PRRs, inhibit IFN induction, and block IFN signaling cascades. Finally, we will discuss the potential of harnessing innate immune machineries for the control of enteric coronavirus infection, and implications of this knowledge on development of immune modulators for effective vaccination against these two pathogens.

## PEDV and PDCoV Biology

**Figure 1:**
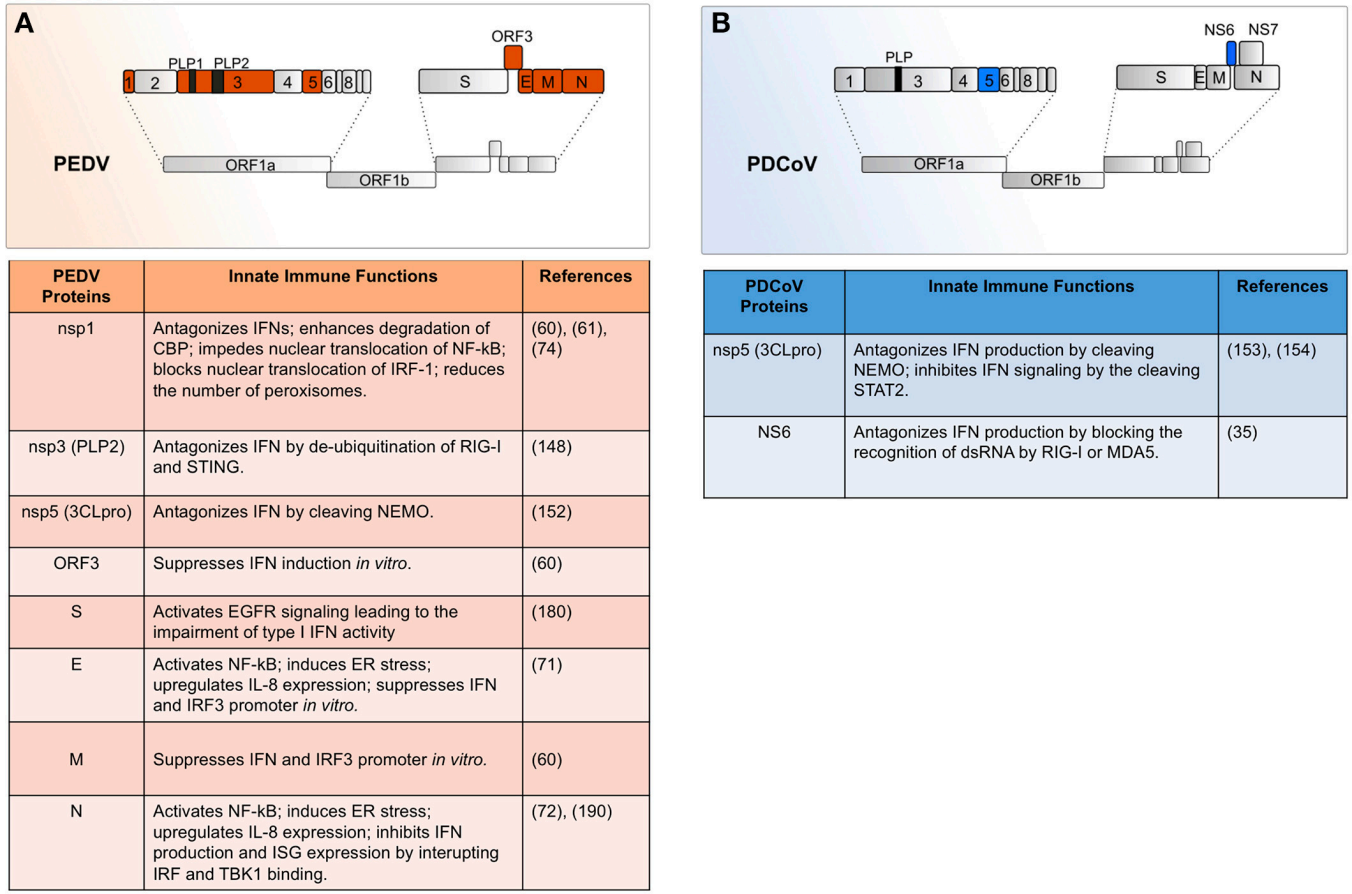
Both PEDV and PDCoV are enveloped viruses with single-stranded positive-sense RNA genomes of ~28 and 26 kB in length, respectively. **(A)** Within the genome of 28 kB of PEDV, so far seven encoded proteins have been shown to implicate in the innate immune modulation (highlighted here in orange). The first two of the seven open reading frames (ORFs) encode replicase 1a and 1b, respectively which are the two polyprotein precursors of 16 non-structural proteins. The rest of ORFs encode four structural proteins which constitute the virion, and one accessory protein namely ORF3. The structural protein S, E, M, and N as well as ORF3 accessory protein are implicated in the innate immune modulation and suppression (See texts for details). **(B)** Similar to PEDV, the replicase polyprotein 1a (pp1a) and pp1b of PDCoV are also cleaved by virus-encoded proteases into 16 non-structural proteins. The ensuing ORFs; however, encode four structural proteins, as well as two non-structural (NS) accessory protein NS6, NS7, and NS7a. So far, two virus-derived proteins with proposed roles as the innate immune antagonists have been reported (highlighted here in blue).

Both PEDV and PDCoV are enveloped viruses with single-stranded positive-sense RNA genomes of ~28–26 kB in length, respectively (2, 24) Their genome organization is depicted in Figure 1. Open reading frame 1a (ORF1a) and ORF1b of both viruses encode two polyprotein precursors, pp1a and pp1ab, which are cleaved by the papain-like protease (PL-pro) and a serine type 3C-like protease (3CLpro) (25) to give rise to non-structural proteins (nsp) 1–16 for PEDV and nsp1–15 for PDCoV (26–28). Many of the individual nsps interact to form the replicase-transcriptase complex (RTC) responsible for viral RNA replication and transcription of sub-genomic RNAs. In addition to these replication functions, some coronavirus nsps are also involved in antagonizing host innate immune responses.

Both PEDV and PDCoV possess four structural proteins, namely spike (S), envelope (E), membrane (M), and nucleocapsid (N). Each virus has a unique set of accessory proteins, however. PEDV has only one accessory protein, ORF3 (29), whereas the PDCoV genome encodes non-structural (NS)6, NS7, and NS7a accessory protein (30, 31). Although distributed widely both within and between structural genes, the location and function of coronavirus accessory protein genes are species-specific (32). In fact, coronavirus accessory proteins possess diverse functions, including modulating viral pathogenicity (33), inducing cell death (34), or antagonizing the IFN system (35–37).

PEDV and PDCoV primarily target the GI tract of pigs, although PEDV has also been found to infect alveolar macrophages of the respiratory tract resulting in pneumonic lesions (38). While the fecal-oral route accounts for the main means of PEDV and PDCoV transmission, vomitus, and contaminated fomites such as transport trailers and feed may also be points of viral transmission (39, 40). Upon host entry via the oral route, porcine coronaviruses bind to surface receptors on susceptible cells, primarily villous epithelial cells of the small intestine brush border (38, 41, 42). In swine, porcine aminopeptidase N (pAPN) which is highly expressed in the small intestinal mucosa was implicated to play a critical role in the target cell infection of PEDV and PDCoV (43, 44). Following cell entry, porcine coronaviruses, similar to most of CoV, initially form double-membrane vesicles (DMVs) where replication/transcription probably takes place, assemble in the rough endoplasmic reticulum and the large virion containing vacuoles (LVCVs), and are transported via the Golgi apparatus for release by budding from the surface membrane of the infected cells (26, 45–48). Infected villous cells are then destroyed, leading to reduction, and shortening of the villi.

Because both PEDV and PDCoV target villous enterocytes of the porcine GI tract, establishment of a productive infection requires both penetrating the heavily guarded mucosal barriers and circumventing the host’s robust and rapid innate immune response. Although many comprehensive reviews have described how other coronaviruses such as the severe acute respiratory syndrome (SARS)-CoV (49–51), and Middle Eastern respiratory syndrome (MERS)-CoV (52, 53) interact with components of innate immunity, knowledge about how PEDV, and PDCoV antagonize host innate immune responses has only started to emerge. Furthermore, due to the challenges in propagating field isolates in a biologically relevant cell culture system and difficulties in viral genome manipulation, the mechanisms behind porcine enteric virus pathogenesis remain largely unknown.

## *In vitro* Models for PEDV and PDCoV Infection: Cell Lines and Primary Cells

Cell lines provide invaluable information on viral pathogenesis and its interplay with the innate immune response. The lack of suitable cell lines is therefore one of the major impediments to progress in the field. For the study of porcine enteropathogenic viruses, for instance, many of the most widely used cell lines are not even derived from natural target cells, namely enterocytes of intestinal villi. As a case in point, the staple cell line for PEDV propagation has been Vero, derived from the kidney of an African green monkey, since the process was first described by Hofmann and Wyler (54). The use of Vero cells, however, is limited to the propagation of cell-adapted PEDV strains. The success rate of expanding new variant and field-isolated PEDV in Vero cells is rather low and often comes at the cost of gradual loss of infectivity during passaging (55). While being permissive to PEDV propagation and replication, these cells have a major deletion in the type I IFN gene cluster, resulting in IFN deficiency (56–59) and thus rendering them unsuitable for studying viral modulation of innate immune responses.

Cell lines such as MARC-145 (African green monkey kidney), LLC-PK1 (porcine kidney), and ST (swine testicle) may be more appropriate for studying PEDV-mediated innate immune modulation. Zhang et al. examined various cell lines for PEDV susceptibility and discovered that the IFN-competent MARC-145 cells were also permissive for PEDV infection, exhibiting cytopathic effects (CPE) and infection foci staining comparable to infected Vero cells (60). Using these cells, they were able to demonstrate the suppression of type I IFN production and degradation of CREB-binding protein (CBP) by PEDV. They also used LLC-PK1 and ST cells to investigate the role of PEDV nsp1 protein in the inhibition of early NF-κB activation (61).

Other immortalized cell lines permissive for PEDV include PK-15 (porcine kidney), Huh-7 (human liver), MRC-5 (human lung), and Tb1-Lu (bat lung) cells, which were used to examine PEDV receptor usage and cell entry (62). A comprehensive list of both traditional and newly established cell lines currently being tested or permissive for PEDV replication can be found in a recent review by Teeravechyan et al. (63). These cells possess a variety of phenotypes, however, and will need to be carefully vetted before use in studying innate immune responses to PEDV.

Only two immortalized cell lines of swine origin, namely ST and LLC-PK1, are known to be permissive for PDCoV and used for its isolation and propagation (64). At 2 days post-inoculation, PDCoV-infected LLC-PK1 and ST cells become enlarged and rounded, characteristics of PDCoV-associated CPE. While the presence of trypsin in maintenance media helps to improve PDCoV propagation in the LLC-PK1 cell line, its absence does not completely abrogate virus propagation, unlike for ST cells. Additionally, cell culture media supplemented with pancreatin and/or small intestine content (SIC) solution extracted from healthy uninoculated gnotobiotic pigs supported PDCoV propagation in both LLC-PK1 and ST cells. LLC-PK1 has also been used to demonstrate PDCoV antagonism of various host innate immune components (65, 66).

Although the use of these cell lines has provided invaluable information about the interaction between these two enteric coronaviruses and their hosts, it may not yield relevant biological information consistent with *in vivo* PEDV and PDCoV infection because these cells are not derived from pig intestinal epithelial cells (IEC), the known target cells of both porcine coronaviruses. In recent studies, immortalized IECs have been derived by the introduction of the human telomerase reverse transcriptase (hTERT) gene into the neonatal-derived small intestinal epithelial cells (67) and used by many groups for PEDV propagation (68–72). However, only IPEC-J2, a porcine jejunal cell line derived from a neonatal pig, has been used to study how PEDV antagonizes host cell antiviral activity (73). The use of IPEC-J2 cells could provide more biologically relevant information when investigating the pathogenesis of PEDV infection; however, others found that these cells were not always susceptible to PEDV (32). In their study, Zhang et al. claimed that IPEC-J2 cells, in addition to its relative non-permissiveness to PEDV infection, were actually heterogeneous, and that the infection rate achieved by this cell line was extremely low. A new cell line, IPEC-DQ, was thus sub-cloned and characterized for PEDV propagation (74). IPEC-DQ cells were found to support efficient and productive infection of PEDV. Furthermore, due to their ability to express type III IFNs, IPEC-DQ could potentially be used as a suitable cell model for the study of gut innate immunity and its modulation by PEDV. Nevertheless, immortalization and transformation of primary cells may affect cellular antiviral signaling, possibly resulting in misrepresentation of *in vivo* innate immune responses. In fact, a number of cellular pathways regulating IFN-stimulated genes and antiviral defense are closely linked to cellular tumor suppression activity, including anti-proliferative, pro-apoptotic, and pro-inflammatory responses (75). Accordingly, the antiviral responses observed in immortalized IEC or IPEC cells, despite being of porcine intestinal epithelial cell origin, should be further compared to those in primary IECs.

Consistent with this idea, primary porcine IECs were recently isolated and used to propagate PEDV (76). For the first time, primary porcine IECs were used as a model to study the interplay between molecular mechanisms of PEDV infection and the host innate immune response, focusing on the potential mechanism of PEDV-mediated NF-κB activation in particular. Although porcine IECs are the ideal cell type for PEDV and PDCoV research and representative of target cells *in vivo*, these cells are difficult to procure, have a short life span and, unlike immortalized cell lines, could contain a mixed population of different cell types. Ectopic or stable expression of exogenous genes in primary cells is also very difficult due to differences in doubling time and life span of each primary cell type, making clonal selection virtually impossible. Another important technical reason that limits the use of primary IECs in PEDV and PDCoV research is their hypersensitivity to trypsin required for enteric coronavirus propagation in *in vitro* culture (54).

All things considered, porcine IEC-derived immortalized cell lines remain the optimal *in vitro* models for studying the innate immune response to PEDV and PDCoV infection, balancing ease of use with a close approximation to *in vivo* target cells.

## Overview of Innate Immune Responses to Viral Infection

**Figure 2:**
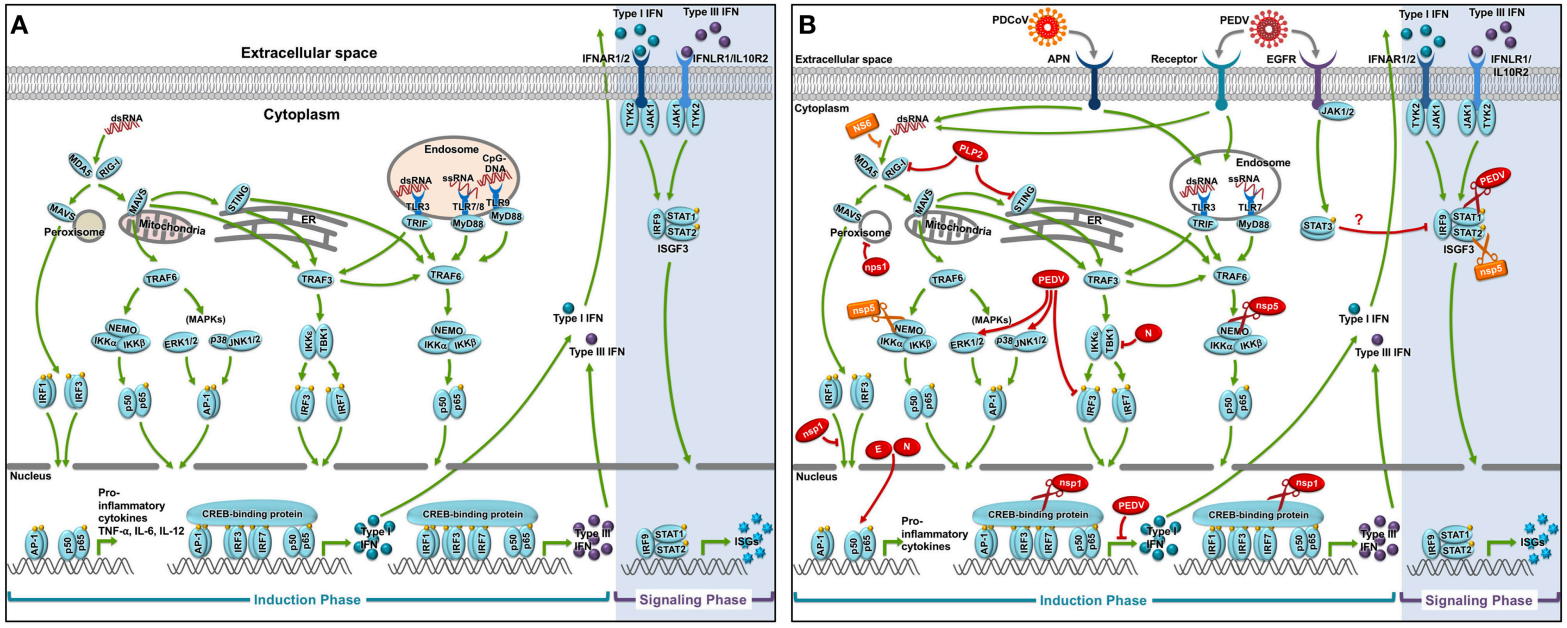
Innate immune signaling pathways and antagonism by PEDV and PDCoV proteins. Following the cellular receptor-mediated entry into the target cells, the genomic RNAs of both PEDV and PDCoV are released into the cytosol by viral-host membrane fusion. During the cytokine induction phase, the presence of the virus-derived RNA genome as well as other replicative RNA intermediates are sensed by both the endosomal TLRs (TLR3, 7/8) and cytosolic RLRs (RIG-I and MDA5). The recognition of the virus-derived RNAs by these receptors triggers a cascade of signaling molecule activation leading to a nuclear translocation of the key transcription factors including NF-κB, IRF1, IRF3, and IRF7. Inside the nucleus, the binding of these transcription factors to their respective PRD regions drives the production of type I and type III IFNs, and pro-inflammatory cytokines which are then secreted into the extracellular space. Subsequently, in the signaling phase, the engagement of both type I and III IFNs to their cognate receptors in both autocrine and paracrine manner induces the activation of JAK/STAT pathway leading to nuclear translocation of the ISGF3 complex as well as the subsequent production of the interferon stimulating genes (ISGs) **(A)**. These ISGs confer the cells with an anti-viral state. In order to ensure the establishment of a successful infection, both PEDV and PDCoV either produce viral proteins (shown in red for those of PEDV and in yellow of PDCoV) to directly antagonize various critical steps of both IFN induction and signaling or affect indirectly the host cell anti-viral signaling cascades **(B)**.

Mammalian hosts are equipped with innate immune mechanisms which launch immediate responses against viral infection. This first line of defense prevents the establishment of successful infection and systemic spread, and in many cases, destroys invading viruses even before the adaptive arm of host immunity is mobilized. A schematic diagram of the host innate immune signaling pathways is depicted in Figure 2A.

Upon viral infection, infected host cells can sense the presence of both viruses and viral products by three main classes of host PRRs (77). These are the endosomal toll-like receptors (TLRs), the cytoplasmic retinoic acid-inducible gene I (RIG-I)-like receptors (RLRs), and the nucleo-oligomerization domain (NOD)-like receptors (NLRs).

TLRS are found both in the endosomal and cell surface membranes. While signaling mediated by most cell surface-localized TLRs induce only pro-inflammatory cytokine responses and not IFN expression, activation of endosomal TLRs and a plasma membrane-resident TLR4 may lead to both (78). Among the TLRs characterized to date, those localized to endo-lysosomal compartments include TLR3, TLR7, TLR8, and TLR9, with each detecting distinct forms of viral nucleic acids. On the other hand, RLRs and NLRs are cytoplasmic sensors. Three types of RLRs have been identified—retinoic acid-induced gene I (RIG-I), melanoma differentiation associated gene 5 (MDA5), and laboratory of genetics and physiology 2 (LGP-2) (79). NLRs are mostly associated with recognition of bacterial PAMPs (80, 81) and will not be discussed further in this review.

While both TLRs and RLRs are capable of recognizing viral PAMPs, particularly double-stranded RNA (dsRNA), they utilize different adaptor proteins to initiate their signaling cascade. The TLR signal transduction pathways are dependent on either myeloid differentiation primary response 88 (MyD88) or TIR domain-containing adapter-inducing interferon-β (TRIF) (78, 82). RLRs, on the other hand, utilize the mitochondrial activator of virus signaling (MAVS/IPS-1/VISA/CARDIF) as the essential signaling adaptor protein (79, 83). TANK-binding kinase 1 (TBK1) and inhibitor of nuclear factor kappa-B kinase subunit epsilon (IKKε) interact to relay signals to the critical transcription factors interferon regulatory factor 3 (IRF3) and NF-κB, leading to their phosphorylation and nuclear translocation (84). Activation of these transcription factors as well as AP-1 then initiate transcription of type I IFNs IFN-α and IFN-β.

The induction of IFN-α and IFN-β is one of the hallmarks of the host innate immune responses against invading viral pathogens. These secreted soluble factors represent a family of antiviral cytokines which, upon binding to their surface heterodimeric receptor (composed of the IFNAR1 and IFNAR2 subunits), leads to the activation of the receptor-associated tyrosine kinases, Janus kinase 1 (JAK1), and tyrosine kinase 2 (Tyk2). These kinases phosphorylate the signal transducer and activator of transcription 1 (STAT1) and STAT2 (85). The phosphorylated STAT1/STAT2 heterodimer then translocates into the nucleus, where it interacts with IRF9 to form IFN-stimulated gene factor 3 (ISGF3). This, in turn, binds to IFN-stimulated response elements (ISRE) in gene promoter regions, leading to the expression of antiviral effectors known as IFN-stimulated genes (ISGs) (86–88). ISGs function to restrict viral replication, modulate other aspects of innate immunity and prime the adaptive immune response (89).

Although type I IFNs and ISGs are the main host antiviral components and act as the first line of defense against viral infection, type III IFNs (such as IFN-λs) have recently been described to contribute to the host antiviral state as well as induce ISG expression (90). Type III IFNs share significant functional similarities with type I IFNs. All IFN-λs bind a heterodimeric IFN-λ receptor complex (IFNLR) for signaling (91). While previous *in vitro* studies demonstrated that types I and III IFNs are co-produced in response to viral infection or the presence of PAMPs, particularly nucleic acids which trigger both extracellular and intracellular sensors, more recent *in vivo* experiments support the observation that mucosal infections appear to trigger predominantly IFN-λ expression and a low level of IFN-β (92, 93). Consistent with this, epithelial cells which protect the GI tract mucosal lining were found to be the main source of IFN-λ production during enteric virus infections (94–96). Indeed, IFN-λ is known to be critical in controlling infection of epithelial cells by various enteric viruses, including norovirus, reovirus, rotavirus, adenovirus, and murine cytomegalovirus (97). Selectively high expression of IFNLRs on IECs in the GI tract argues for the indispensable contribution of type III IFNs to the initiation of early antiviral responses in this organ (95).

Although the induction of types I and III IFN pathways involves a great deal of overlap in the signaling cascade leading to establishment of a cellular antiviral state, there are still differences in transcription factor requirements (90, 98). While IFN regulatory factor (IRF)-3,-7, and NF-κB are essential components for induction of both types I and III IFNs, IRF1 seems to play a unique role in the type III IFN pathway (99). Additionally, unlike RLR-mediated type I IFN induction, intracellular sensors of type III IFN depend largely on peroxisomal MAVS for a rapid but rather short-lived induction of IFN expression (100, 101).

Successful establishment of viral infections generally require the ability to evade, antagonize, or subvert innate immune responses. Indeed, previous studies have shown that PEDV infection inhibits type I IFN induction in several cell types, such as MARC-145 or porcine IECs (60, 76), and exhibits relative resistance to IFN-α by inducing the proteasome dependent degradation of STAT1 (73). These observations suggest that this virus has developed strategies to prevent the biological activities of IFNs. Similarly, PDCoV has also demonstrated antagonism of IFN production in cell culture (66). The antiviral effects of IFN-λs may also play a crucial and as-yet underappreciated role in both PEDV and PDCoV infection. The mechanisms by which PEDV- and PDCoV-encoded proteins modulate components of the IFN induction pathways is summarized in Figure 2B and will be discussed later in this review.

## Cellular Innate Immune Responses to PEDV and PDCoV

In addition to type I and III IFN induction, viral infection also results in the recruitment of innate immune cells such as DCs, macrophages, and natural killer (NK) cells to the site of infection (102). These innate immune cells not only provide immediate counterattacks against invading viruses, but also present foreign antigens to T cells and prime adaptive immune responses via cytokine secretion (103).

DCs and macrophages are the two most prominent cellular components of innate immune responses. Given the essential role of both cell types in professional antigen presentation and immune cell activation, it is important to gain a better understanding of the interaction of coronaviruses with these professional APCs. A study with SARS-CoV demonstrated that although neither macrophages nor DCs were productively infected, many phenotypic changes in cell viability, expression of MHC class II, CD40, CD83, and CD86, and the ability to stimulate T cell proliferation were observed in these cells upon exposure to live virus (51). Macrophages were both refractory to such stimuli and displayed diminished phagocytic activity whereas DCs were observed to display upregulated MHC class II, CD40, CD83, and CD86 expression. As a consequence, these SARS-CoV-primed DCs were able to efficiently stimulate allogenic T cell proliferation.

For PEDV, however, the data are still conflicting regarding the susceptibility of DCs to PEDV infection (104, 105). *In vitro*, the classical PEDV strain CV777, in contrast to SARS-CoV, could productively infect both immature and mature monocyte-derived dendritic cells (Mo-DCs) leading to the enhanced ability of Mo-DCs to sample antigens and present them to T cells for T-cell activation (104). Interestingly, Gao et al. also observed that immature Mo-DCs were more susceptible than mature Mo-DCs to infection by CV777, possibly due to their higher rates of endocytosis and aminopeptidase N (CD13) expression. Furthermore, infected immature Mo-DCs up-regulated CD1a, CD80/86, and SLA-II-DR, which have been shown to enhance the cells’ antigen presentation function (106, 107). Up-regulation of CD1a, CD80/86, and SLA-II-DR was observed to a lesser extent for infected mature Mo-DCs. When CV777 infection was studied *in vivo*, the virus was found to rapidly infect intestinal DCs (104). Based on these observations, Gao et al. suggested that both Mo-DCs and intestinal DCs play a role in priming and promoting an effective response during PEDV CV777 infection.

In contrast, a recent study by Wang et al. showed that PEDV failed to undergo a productive replication in porcine Mo-DCs (105). In spite of this, infection activated transcription of type I IFN and chemokine interferon-inducible protein-10 (IP-10). Unfortunately, the molecular mechanisms by which PEDV triggered type I IFN and chemokine IP-10 expression in the absence of active virus replication and the implication of these cytokines in PEDV pathogenesis and immunity remain to be determined.

DCs can also be exploited and hijacked by PEDV as vehicles for viral transmission. A recent study implicated porcine bone marrow-derived DCs in the dissemination of PEDV from the swine nasal cavity to intestinal mucosa (108), supporting the hypothesis that PEDV could be spread from infected pigs through airborne transmission. This study also verified that PEDV could enter porcine nasal epithelial cells (NECs) via their apical side adjacent to the nasal mucosa and establish transient infection within the nasal cavity. Submucosal DCs residing near infected nasal epithelial cells (NECs) then take up PEDV from the lumen across the nasal mucosa via their extended cellular processes. Despite the lack of active viral replication in these DCs, these virus-loaded DCs could subsequently transfer the viruses to T cells which then enter peripheral blood. These recirculating T cells finally shuttle the viruses to the intestinal epithelium, leading to typical PEDV symptoms. The utilization of DCs, which are widely distributed in the mucosal lining of various tissues, by PEDV as carriers to overcome mucosal barriers and disseminate throughout the body is reminiscent of how many other viruses establish a foothold upon entering the host body (109–112).

Similarly, the permissiveness of macrophages to PEDV is still unclear. Lee et al. showed that viral antigen could be detected in lamina propria-resident macrophages of infected pigs (113). There has also been one report of PEDV infection in alveolar macrophages, resulting in pneumonic lesions (38). Despite these observations, detection of viral replication in these cells has yet to be reported.

NK cells are responsible for cytotoxicity-mediated killing of virus-infected cells and are a major source of IFN-γ, TNF-α, GM-CSF, and other cytokines and chemokines (114, 115). The observation that PEDV-infected neonatal and nursing piglets with more severe symptoms possessed lower NK cell numbers suggest for a potential role for NK cells in the host antiviral response to these pathogens (23). In their study, Annamalai et al. showed that both quantitative and qualitative variation of NK cell properties can be observed in response to PEDV infection in suckling and weaned pigs. In uninfected animals, suckling pigs, which are much more susceptible to PEDV, have drastically lower NK cell numbers than weaned pigs in both the blood and the ileum. Upon infection, significant IFN-γ production is observed from weaned pig NK cells, unlike those of suckling pigs. Strangely, frequencies of NK cells in the blood were found to be higher than in the ileum, the primary site of PEDV infection, and became even more disproportionate during the course of infection. In addition, serum levels of IFN-α, IL-12, and TNF-α peaked at an earlier time point in infected suckling pigs, indicating faster progression of disease, when compared to those of weaned pigs, and coincided with viral shedding and onset of diarrhea in both groups of pigs. Due to these disparate observations, it remains to be seen whether NK cells play a direct antiviral function during PEDV infection.

It is worth pointing out that the increase in serum pro-inflammatory cytokine and chemokine levels in both PEDV infected suckling and weaned pigs mentioned above could reflect the outcome of the simultaneous induction of the key components of the mitogen-activated protein kinase (MAPK) cascade including Erk1/2 and JNK/p38 (116, 117), and the activation of NF-κB pathway (72, 118) during the PEDV infection. As shown in the context of other pathogenic infections (119, 120), the concurrent activation of both MAPK components and the transcription factor NF-κB during PEDV infection might lead to the upregulation of both pro-inflammatory cytokines and chemokines. While the PEDV N protein was described by Xu et al. as a virus-derived intermediate responsible for triggering the NF-κB pathway activation, the exact mechanism of MAPK activation following PEDV infection is still not determined. It was demonstrated, however, that the PEDV-mediated MAPK activation enhanced the viral replication. These findings argue for the notion that PEDV, to its advantage, could manipulate host intracellular processes including the stimulation of the MAPK cascade and the NF-κB pathway.

For PDCoV infections, information remains extremely limited regarding the interplay with the cellular innate immune response. There are some reports describing infiltration of macrophages, lymphocytes, eosinophils, and neutrophils in the lamina propria of the small intestine during infection (14, 121, 122). However, there is still a lack of any evidence regarding whether these innate immune cells actually engage in anti-PDCoV responses and what such responses might be.

## PEDV and PDCoV Antagonists of Innate Immunity

### Non-structural Proteins

The coronavirus nsps have been shown to be involved mainly in viral RNA synthesis (123–127). Nevertheless, nsp1, 3, 5, 7, 14, 15, and 16 have been observed to play additional roles in host immune modulatory functions (50, 60, 128–136). Due to the early expression of these non-structural proteins, their ability to suppress innate immune responses provides invading viruses with the opportunity to replicate and establish a productive infection.

#### PEDV nsp1

Nsp1 is only present in alpha- and betacoronaviruses (2, 137). Despite its relatively small size at 110 amino acids in length, nsp1 shows great genetic sequence variation among alphacoronaviruses (138, 139) which may account for its functional versatility and the ability to interact with a number of host innate immune signaling molecules. Like the SARS-CoV nsp1, PEDV nsp1 was shown to be a potent IFN antagonist interfering with both IRF- and NF-κB-mediated induction of type I and III IFNs (60, 61, 74). These effects occur through either enhancing degradation of or inhibiting nuclear translocation of host key signaling molecules involved in *IFN* gene activation such as the CREB-binding protein (CBP) (60), which forms part of the promoter-binding enhanceosome complex with NF-κB, AP-1 [a complex of activating transcription factor 2 (ATF2) and JUN], and homodimers or heterodimers of IRF3 and IRF7 (140). PEDV nsp1 promotes proteasome-mediated degradation of CBP, which renders *IFNB* gene transcription induction less effective as binding of the enhanceosome to the *IFNB* gene promoter is more stable than any of its components alone (141).

PEDV nsp1 is also known to impede nuclear translocation of NF-κB, affecting not only production of IFN-β but also proinflammatory cytokines such as TNF-α, IL-1β, IL-6, IL-15, and IL-17 (61). This occurs through inhibition of IκBα phosphorylation and its subsequent ubiquitin-mediated degradation, which are required for NF-κB transport to the nucleus where it can then bind to target sequences and initiate transcription (142–144). The activity of nsp1 against IκBα was also found to block nuclear translocation of the p65 (also named RelA) subunit of NF-κB, preventing the dimer formation between RelA and the p50 subunit of NF-κB (p50/RelA) important for NF-κB signaling. Taken together, the PEDV nsp1-mediated inhibition of type I IFNs and pro-inflammatory cytokines through suppression of NF-κB activity argue for the antiviral potential of these cytokines during the early stage of PEDV infection.

PEDV nsp1 also modulates type III IFN responses in IECs (74). In PEDV-infected IPEC-DQ, LLC-PK1, and MARC-145 cells, nsp1 was observed to block nuclear translocation of IRF1 and reduced the number of peroxisomes, where peroxisomal MAVS link RLR signaling to type III IFN induction. Furthermore, the observed reduction in the number of peroxisomes and IRF1-mediated IFN-λ suppression were dependent on the conserved amino acid residues of PEDV nsp1. This intriguing insight into PEDV interference with the type III IFN pathway should pave the way for future studies elucidating the as-yet underappreciated role for IFN-λ in the control of porcine enteritis coronavirus infection.

#### PEDV nsp3

In coronaviruses, the PLpro domain of nsp3 and the 3CLpro domain of nsp5 facilitate viral replication by processing pp1a and pp1ab polyprotein precursors into nsps. It has also been demonstrated that many human and animal coronavirus-derived proteases mediate negative regulation of host antiviral innate immunity. Previous studies showed that the PLpro of the human coronaviruses SARS-CoV and NL63-CoV antagonize innate immune induction of type I IFNs via de-ubiquitination/deISGylation of NF-κB signaling molecules, inhibition of IRF3 activation and nuclear translocation, and blocking ubiquitination of STING and disrupting its dimerization (145–147). Xing et al. recently demonstrated that the PEDV PLpro domain, PLP2, also interferes with RIG-I- and STING-mediated type I IFN activation (148) through de-ubiquitinating activity, preventing the post-translational modification of RIG-I by Lys63-linked ubiquitination that is essential for RIG-I-mediated signaling (149). Similar to RIG-I, ubiquitination of STING is critical for expression of downstream antiviral genes (150, 151). Accordingly, PEDV PLP2-mediated de-ubiquitination of RIG-I and STING leads to abrogation of downstream signaling and inhibition of type I IFN expression.

#### PEDV and PDCoV nsp5

The 3CLpro of both PEDV and PDCoV, encoded by the nsp5 gene, have also been shown to antagonize innate immune signaling through proteolytic cleavage of host key signaling molecules (152–154). Wang et al. provided evidence that PEDV nsp5 disrupts type I IFN signaling by cleaving a critical adaptor protein, the NF-κB essential modulator (NEMO; also called IKKγ), which bridges the NF-κB and IRF signaling pathways by triggering NF-κB and IRF3 nuclear translocation and eventual induction of IFN-β production (79, 155, 156). Highly conserved histidine 41 (His41) and cysteine 144 (Cys144) of PEDV nsp5 were identified as the catalytic dyad responsible for protease activity of nsp5 and suppression of IFN-β induction. Similarly, PDCoV nsp5 also inhibits IFN-β production through the cleavage of NEMO, and its protease activity dominates its ability to antagonize IFN-β induction (154). Interestingly, nsp5 of both PEDV and PDCoV target the glutamine 231 (Q231) of NEMO, suggesting that nsp5 proteolytic cleavage of NEMO is highly conserved and specific in both coronaviruses. This is in contrast to cleavage of NEMO by 3C or 3C-like proteases of other viruses such as the foot-and-mouth disease virus (FMDV), hepatitis A virus (HAV), and porcine reproductive and respiratory syndrome virus (PRRSV) which cleave NEMO at Q383, Q304, and E349, respectively (157–159).

In addition to PDCoV nsp5 inhibition of IFN-β induction, the protein also antagonizes type I IFN signaling downstream of IFN Receptors by targeting the JAK-STAT pathway (153). Zhu et al. demonstrated that PDCoV nsp5 cleaves STAT2, one of the components of ISGF3, disrupting the function of the ISGF3 complex in initiating the ISG-mediated antiviral state. They also discovered that STAT2 cleavage activity is probably unique to PDCoV nsp5, as no other coronavirus nsp5, including those from PEDV, TGEV, SARS-CoV, MERS-CoV, HCoV-229E, HCoV-OC43, and HCoV-NL63, could catalyze STAT2 cleavage. Interestingly, while PEDV infection leads to no cleavage of STAT2, it is capable of both promoting degradation and interrupting activation of STAT1 without inhibiting STAT1 transcription (73). In their study, Guo et al. showed that PEDV infected VeroE6 and IPEC-J2 cells had diminished STAT1 levels when compared to those in uninfected cells. Unlike PDCoV-mediated STAT2 cleavage, PEDV infection-induced STAT1 degradation relies on the ubiquitin-proteasome system. Furthermore, although STAT1 degradation was confirmed in PEDV-infected cells, it remains to be seen whether any PEDV proteins are the main culprits responsible for this process.

### Accessory Proteins

Although a great deal of evidence supports the notion that coronavirus accessory proteins function in host–pathogen interactions and mediate viral pathogenesis during coronavirus infection *in vivo* (33, 160), emerging studies have begun to shed light on the interplay between these accessory proteins and the host innate immune system, arguing for their possible role in the regulation of host antiviral responses (161–164). Notable immune regulation activity has been reported for coronavirus accessory proteins 3b (p3b), 6 (p6), and 9b (p9b) of SARS-CoV translated from ORF3, ORF6, and ORF9, respectively; ORF4a and ORF4b of MERS-CoV; and ns2 of mouse hepatitis virus (MHV) (36, 163–170).

The possible role of PEDV’s sole accessory protein, ORF3 in host innate immune regulation remains as enigmatic as its role in pathogenesis. So far, only one study has directly implicated the PEDV ORF3 protein in suppression of type I IFN induction *in vitro*, with overexpression of ORF3 resulting in anti-IFN activity as assayed by a luciferase reporter assay (60). Many groups have, however, proposed a role for ORF3 in virus growth and replication (171–175), as some strains of cell-adapted PEDV display either internal truncation or amino acid sequence variation in the ORF3 gene (172, 175, 176). As these changes are generally seen after adaptation to IFN-deficient Vero cells, there is a possibility that future work may link ORF3 more strongly to modulation of the innate immune response.

For PDCoV, three accessory proteins have been identified, namely NS6, NS7, and NS7a (11, 30, 31). The function of these proteins in viral replication, pathogenesis, and immune regulation remain mostly unclear. NS6 has recently been shown to antagonize the host innate immune response (35). Similar to SARS-CoV accessory proteins ORF6 and ORF9b, PDCoV NS6 was identified as being virion-associated and an inhibitor of IFN-β expression (30, 163, 177, 178). NS6 acts by blocking the recognition or binding of dsRNA by RIG-I or MDA5, likely by directly binding to either the C-terminal domain (CTD) of RIG-I or the helicase domain and CTD of MDA-5, or both, as its ability to bind to viral RNA was not observed (35).

### Structural Proteins

The C-terminal end of the PEDV and PDCoV genomes encode the structural proteins S, E, N, and M. Among these, ectopic expression of PEDV E, N, and M has been shown to antagonize the IFN-β and IRF3 activity (60). Recent successive publications by Xu et al. also provided details on host cell responses to the presence of PEDV E, M, and N proteins, specifically their effect on cell growth and the cell cycle, ER stress, NF-κB activation, and IL-8 and Bcl-2 expression (71, 72, 179).

#### PEDV S

In addition to its indispensable role in virus entry into the target cell through receptor binding and subsequent fusion of the viral and cellular membranes, a recent study by Yang et al. revealed the newly discovered role of the PEDV S protein in the impairment of the anti-PEDV activity of type I IFN (180). Yang et al. demonstrated that PEDV (both live and killed) through direct interaction between the S protein and epidermal growth factor receptor (EGFR), induced EGFR activation which, in turn, augmented PEDV infection. They further demonstrated that, by acting via one of its downstream signaling pathways, namely JAK2-STAT3, the EGFR activation helped to enhance, and facilitate PEDV replication. It is worth pointing out that while the roles of EGFR signaling in the cell to cell communication as well as the transformation of various types of cancer were well documented (181, 182), its involvement in facilitating PEDV infections through the suppression of type I IFN-mediated antiviral response is in accordance with previous findings described in studies of other viruses (183–185). Although direct binding of the PEDV S protein to EGFR is sufficient to trigger both the EGFR activation and the attenuation of type I IFN activity, further studies are still needed to identify the underlying mechanisms leading to the crosstalk between both EGFR and type I IFN signals.

#### PEDV E

PEDV E protein is a small 7-kDa membrane protein encoded by the *E* gene which is located downstream of PEDV ORF1a and ORF1b. The protein plays an important role during coronavirus budding (29). Xu et al. demonstrated that the E protein could induce ER stress in transfected cells through up-regulation of glucose-regulated protein 78 (GRP78), a marker of ER stress, and activation of NF-κB, coinciding with E protein localization to the ER (71). They also speculated that E protein-mediated activation of NF-κB, in turn, would up-regulate expression of the neutrophil chemotactic factor IL-8 as well as the anti-apoptotic protein Bcl2, contributing to both an inflammatory response and persistent PEDV infection. Whether, the effects above recapitulate what really happens in PEDV-infected IECs is still unknown.

#### PEDV M

Unlike the E protein, PEDV M is equally distributed throughout the whole cell instead of being localized mainly in the ER (179). Compared to overexpression of PEDV structural E protein, which was not found to have any effect on IEC growth and cell cycle, PEDV M altered IEC growth and induced cell cycle arrest in the S-phase via the cyclin A pathway. M protein expression neither promoted IL-8 up-regulation nor NF-κB activation in transfected IECs, probably due to the lack of ER stress-inducing effects.

#### PEDV N

Among the 20 mature proteins encoded in PEDV genome, the N protein is the most abundant protein in virus-infected cells and acts as a multifunctional protein involved in viral genome organization, virus assembly, cell cycle regulation, apoptosis induction, host stress response, and translational shutoff (186, 187). Similar to the E protein, PEDV N is also localized to the ER. ER subcellular localization of both E and N, but not M, might account for the ability of both proteins to cause ER stress via IL-8 up-regulation and NF-κB activation (188, 189).

Consistent with the induction of the NF-κB pathway in IECs, Cao et al. recently elucidated a possible mechanism for N protein-mediated NF-κB activation. They demonstrated that over-expressing PEDV N protein in IECs mediated NF-κB activation through TLR2, TLR3, and TLR9 pathways as siRNA silencing of these TLRs dramatically blocked PEDV-induced NF-κB activation (118). Xu et al. also discovered that PEDV N not only induced ER stress via up-regulation of IL-8, Bcl-2, and NF-κB activation, but also inhibited cell growth by prolonging the S phase stage of cell cycle and cyclin A degradation (72). Enhancement of NF-κB signaling is thought to be mediated through the immunodominant central region of N (118).

In contrast to the enhancement of NF-κB signaling observed by both Xu et al. and Cao et al. another recent study showed that PEDV N inhibits IFN-β production and ISG expression by competing with IRF3 for TBK1 binding (190). This interaction inhibited both IRF3 activation and the production of type I IFNs. In accordance with this, PEDV N, along with E and M, was shown to down-regulate both IFN-β and IRF3 promoter activity *in vitro* (60). As previously mentioned, by co-transfecting the plasmids expressing PEDV E, M, and N protein with either pIFN-β-luc or pIRF3-Luc plasmid in Hela cells, Zhang et al. observed a down-regulation of both the IFN-β promoter and IRF3-dependent luciferase activity. The results of this study suggest that the IRF3 signaling pathway is interfered in the suppression of the IFN-β production by PEDV E, M, and N protein. Interestingly, this molecular mechanism is distinct from IFN suppression mediated by the N proteins of other coronaviruses such as SARS-CoV, where N blocks an early step in IFN-β production, probably sensor recognition of viral RNA (191), and MHV, where N targets RNase L activity (192).

The discrepancies observed with the effects of PEDV N on IFN-β and NF-κB induction, as well as the distinct molecular mechanisms it uses to modulate innate immune responses point to the possibility that N protein interacts with multiple host signaling molecules involved in various host signaling pathways. Taken together, the fact that N proteins of different coronaviruses employ different mechanisms to interfere with multiple innate signaling pathways clearly demonstrates the adaptability and coevolution of each coronavirus to a specific host and its associated innate immune pressure.

## Harnessing Innate Immune Anti-viral Activity for PEDV and PDCoV Defense

Harnessing fast-acting antiviral mechanisms of innate immunity has shown promising results in combating a variety of pathogenic viruses. Stimulation of TLR signaling pathways via the use of TLR agonists, for example, has been shown to be an effective means for treating certain viral infections. The use of TLR agonists as innate immune modulators was validated in a study where treatment of vaginal mucosa with a TLR-3 agonist protected mice against genital herpes simplex virus-2 challenge (193). Furthermore, triggering of another endosomal TLR, TLR-7, via systemic administration of a selective TLR7 agonist also elicited anti-hepatitis C virus activity in a clinical setting (194). Direct correlation between such antiviral status and upregulation of IFN production in response to TLR agonist treatment was demonstrated by Cervantes-Barragan et al. where type I IFNs were found to play a dominant role in TLR-mediated antiviral effects (195). In their study, pDCs were identified as the major source of type I IFN when induced through TLR-7 stimulation. While rapid type I IFN production in pDCs was observed following infection with mouse hepatitis virus (MHV), a betacoronavirus, its induction was abrogated in TLR7^−/−^ or MyD88^−/−^ MHV-infected mice, indicating that MHV-mediated type I IFN induction in pDCs was triggered via the TLR7/MyD88 pathway. These observations suggest that the presence of functional type I IFN-producing pDCs in swine GI tracts during exposure to PEDV and/or PDCoV may help to restrict replication of these viruses and thereby regulate the magnitude of clinical severity.

In addition to the induction of antiviral mechanisms via TLR agonists, modulation of the innate immune pathway has also been attempted with synthetic polypeptides harboring innate immune modulatory activities (196). In this study, the recombinant polypeptide N’-CARD-PTD was generated by fusing the N-terminal nuclear localization signal (NLS) of histone H2B, the caspase recruitment domain (CARD) of MAVS, and a protein transduction domain (PTD). Like the TLR agonists discussed previously, these recombinant fusion polypeptides induced strong production of type I IFNs, albeit via a pathway distinct from TLR-mediated signaling. In addition to its potent immunomodulatory function, N’-CARD PTD also augmented immune responses against influenza virus challenge in a mouse model. Whether systemic or local administration of such immunomodulatory polypeptides can restrict PEDV and PDCoV infection in swine is an intriguing question that remains to be explored.

Taking advantage of the knowledge that type I IFNs confer immediate and powerful antiviral responses, several groups of investigators have demonstrated the use of adenovirus type 5 (Ad5) vector-mediated ectopic expression of porcine IFNs or a constitutively active fusion protein of porcine IRF3 and IRF7 [poIRF7/3(5D)] for rapid cross protection against foot-and-mouth disease virus (FMDV) (197–202). While Ad5 vector-based expression of IFNs were found to be potent in the control of FMDV infection, relatively high doses of recombinant Ad5 viruses were required, restricting large-scale application as well as use in emergencies. The efficacy of Ad5 as a biotherapeutic was notably higher when expressing the poIRF7/3(5D) fusion protein, achieving prolonged systemic anti-FMDV activity and upregulation of ISGs in peripheral blood mononuclear cells (PBMC) in inoculated swine (202). The “proof of concept” use of Ad5-poIRF7/3(5D) in the protection of swine against FMDV points to the possibility that GI tract-targeted expression of type I IFNs as well as IRF7/3(5D) could be successfully used to restrict both PEDV and PDCoV. Furthermore, by utilizing similar virus vector-based platforms, regulated, and organ-specific expression of type III IFN could potentially be harnessed to protect against PEDV and PDCoV infection.

The combination of reverse genetics technology to generate recombinant infectious cDNA clones and our growing understanding of viral protein functions in modulating innate immune responses can also lead to the design of more effective candidate vaccines. IFN antagonism by non-structural, structural, and accessory proteins of PEDV and/or PDCoV such as nsp1, nsp3, nsp5, E, M, N, and NS6 can potentially be attenuated by deletion or truncation of these genes, leading to the generation of live attenuated vaccines. Consistent with this idea, disruption or mutation of the SARS-CoV E gene has been a strategy used to generate promising live attenuated SARS-CoV vaccines (203, 204). Furthermore, a TGEV strain with a deleted E gene (TGEV-ΔE) was also put forth as a potential vaccine candidate, demonstrating the ability to target mucosal tissue and induce secretory immunity (205). Despite these observations, the potential of attenuated PEDV and PDCoV carrying a disrupted E gene (or any other genes) as vaccine candidates await further investigation.

Given the critical role of many innate immune mediators, particularly the IFN system, in alleviating severe clinical symptoms, eliminating viral infection, and enhancing vaccine immunogenicity, innate immune machineries may prove powerful tools for tackling a broad range of viral diseases. Indeed, the studies described above accentuate the value and effectiveness of harnessing our knowledge regarding these machineries in antiviral strategies.

## Concluding Remarks

To establish productive infection, invading viruses need to overcome their host’s first line of defense, the innate immune response. Though competent and effective in protecting the host against most microorganisms, this response is still susceptible to antagonism and subversion by pathogenic viruses. Porcine enteritis coronaviruses PEDV and PDCoV, which have recently emerged as important swine pathogens, have evolved strategies to overcome host innate immunity by either avoiding being recognized by PRRs, inhibiting IFN induction, or antagonizing IFN signaling and antiviral effector machinery. While current research has provided copious amounts of invaluable data on how other coronaviruses such as SARS-CoV target molecules involved in the host innate immune response, studies dedicated to host-PEDV and PDCoV interaction have just started to gain traction.

Although both PEDV and PDCoV target pig enterocytes in the intestinal villi, the most common *in vitro* cell culture systems used to study these viruses are not derived from porcine IECs. Both newly derived porcine IEC lines and the availability of three-dimensional intestinal organoids will undoubtedly serve as alternative and more physiologically relevant models for future studies of PEDV- and PDCoV-host interaction. Furthermore, more in-depth study of PEDV and PDCoV pathogenesis *in vivo* will provide less biased data to identify novel host innate immune modulators in the context of viral infection.

Due to their prominent early antiviral function, much attention has been dedicated to type I IFNs. While the defensive roles of type I IFNs in PEDV and PDCoV infection are indisputable, the importance of type III IFNs cannot be ignored. As type III IFNs are selectively expressed by epithelial cells of the intestinal villi in response to viral infection, its roles in anti-PEDV and PDCoV responses warrant a more thorough investigation.

The development of novel and effective mucosal adjuvants and delivery systems may be key to successful PEDV and PDCoV vaccine design for the induction of mucosal immunity, lactogenic immunity and possibly active immunity in newborn piglets. Ultimately, deeper understanding of host early anti-PEDV and PDCoV response will help pave the way to harness our understanding of innate immunity for the development of therapeutic interventions and novel antiviral compounds.

## Author Contributions

SK, ST, and AJ contributed conception and design of the study. SK wrote the first draft of the manuscript. ST, PF, and TC wrote sections of the manuscript. All authors contributed to manuscript revision, read, and approve the submitted version.

### Conflict of Interest Statement

The authors declare that the research was conducted in the absence of any commercial or financial relationships that could be construed as a potential conflict of interest.
